# In vitro measurements of ultrafiltration precision in hemofiltration and hemodialysis devices used in infants, Part 2: Comparison of PrisMax and CARPEDIEM with previous data on NIDUS, Prismaflex and Aquarius

**DOI:** 10.1007/s00467-025-06788-0

**Published:** 2025-07-08

**Authors:** Jean Crosier, Denise Colosimo, Rachel Hansen, Heather J. Lambert, Malcolm G Coulthard, Zaccaria Ricci

**Affiliations:** 1 Great North Children’s Hospital, Queen Victoria Road, Newcastle upon Tyne, NE1 4LP UK; 2 Department of Emergency Medicine and Critical Care, Anaesthesiology and Paediatric Intensive Care Unit, Meyer Children’s Hospital, IRCCS, Florence, Italy; 3 Translational and Clinical Research Institute, Newcastle University, Newcastle upon Tyne, UK; 4 Department of Health Sciences, University of Florence, Florence, Italy

**Keywords:** Infant, Hemodialysis, Hemofiltration, Ultrafiltration, Precision, Aquarius, Prismaflex, PrisMax, NIDUS, CARPEDIEM

## Abstract

**Background:**

We sought to determine in vitro whether the PrisMax and CARPEDIEM hemofiltration and hemodialysis devices can reliably deliver ultrafiltration (UF) control that is sufficiently precise to treat infants.

**Methods:**

We have previously measured the precision of UF control of the Prismaflex, Aquarius and NIDUS devices by in vitro testing with a bag of saline set up as a dummy patient, and comparing the differences between the UF set and displayed by the devices, and the actual fluid removal or addition measured by precise weighing. Here we have tested the PrisMax (updated version of Prismaflex) and the CARPEDIEM using the same method.

**Results:**

The variances of the setting vs. actual errors, and display vs. actual errors after 15 min of ‘treatment’ with the PrisMax and CARPEDIEM were similar, but were significantly larger than in the NIDUS, and much smaller than in the Prismaflex. However, after a 4-h ‘treatment session’, the cumulative errors were still within ± 9 mL for these devices, compared with a maximum error of 2.6 mL in the NIDUS, and a deviation of -37.5 mL in the Prismaflex.

**Conclusions:**

The PrisMax and the CARPEDIEM have adequate precision to be used in infants. The only device with UF error below 3 ml in 4 h is the volumetrically-controlled NIDUS. We recommend that regulatory bodies should introduce UF precision-testing for devices intended for use in infants.

**Graphical abstract:**

**Figure:**
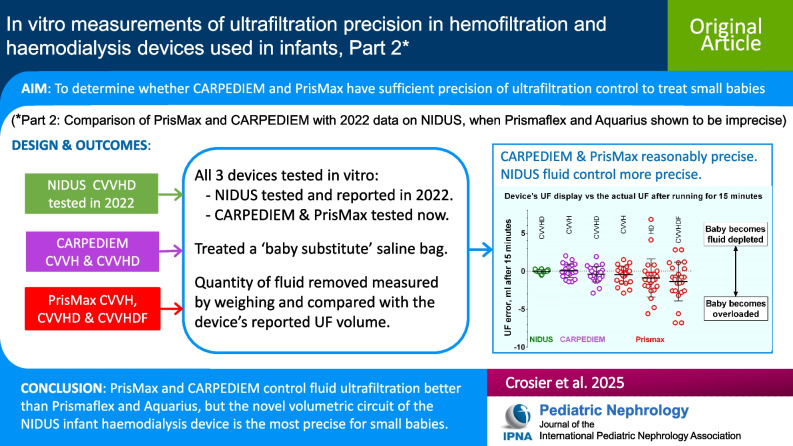
A higher-resolution version of the Graphical abstract is available as  Supplementary information

**Supplementary Information:**

The online version contains supplementary material available at 10.1007/s00467-025-06788-0.

## Introduction

Imprecise control of ultrafiltration (UF) may contribute to clinical instability in small children receiving hemodialysis and/or hemofiltration, and the relevance of such errors may be overlooked by clinicians [1]. There are three main problems – the size of the circuit volume, the inaccuracy of fluid removal and the unreliability of the device display reflecting actual fluid removal. Infants requiring such therapies must be treated with devices that can precisely regulate fluid removal to avoid hypertension and pulmonary oedema from fluid accumulation, and hypovolemic shock from rapid withdrawal. Conventional hemodialysis and/or hemofiltration devices, including Prismaflex and its upgraded version PrisMax, are not licenced for use in children weighing < 20 kg in the US (< 8 kg in Europe) because of a history of imprecision of their UF control [1], but infants continue to be treated off-licence with these and other conventional machines in a relatively unregulated way across the world.

We have previously published in vitro studies of the accuracy and precision of UF control of three devices, none of which were licenced for use in small infants, by setting them each to ‘treat’ a bag of saline instead of a baby, and continuously weighing it to measure the true fluid movements that they generate [2]. The NIDUS is a hemodialysis (HD) device specifically designed for small infants with a novel volumetrically controlled syringe-driven circuit, which has been used in the UK multicentre I-KID clinical study with MHRA approval and on named-patient compassionate grounds outside the study. While it is under consideration for UKCA regulatory conformity, and has been given Humanitarian Use Device (HUD) designation by the FDA, it remains unlicensed for clinical use. The Prismaflex and Aquarius are adult devices that have paediatric lines, but are not licenced for use in children of < 20 kg in the US (< 8 kg in Europe), although they are frequently used for smaller babies off-licence. The NIDUS had UF control that was both accurate (errors close to zero), and precise (variances of < 0.2 mL over 15 min) [2]. By contrast, the Prismaflex had significantly larger UF errors which were present whether the UF target was set at zero or 40 mL/h [2]. It displayed differences between the volumes ‘dialled in’ to the device and those generated (setting vs. actual error), and differences between the achieved UF volume displayed by the device and that generated (display vs. actual error), which had wide variances. This second type of error is likely to be especially misleading because most clinicians will assume that the device’s displayed UF volume is accurate, and will base treatment decisions or UF corrections on that information. The variances for the Aquarius were considerably worse, at approximately twice those recorded for the Prismaflex [2].

At the time of the previous studies, we were not able to access the CARPEDIEM device, which has a miniaturised conventional circuit that is marketed for treating infants between 2.5 and 10 kg, and which was then CE-marked for use in mainland Europe, and is now also FDA-approved for use in the US [3], or the newer PrisMax device. Since then, the paediatric teams from the Meyer Children’s Hospital (where CARPEDIEM and PrisMax are used) and Newcastle Hospitals (where NIDUS and Prismaflex are used) have worked together to test the CARPEDIEM and PrisMax devices using the same protocol as previously, and we compare their UF accuracy and precision values to the previous data from the NIDUS and the Prismaflex.

## Methods

### Experimental design

This was identical to our previous design [2]: a 1L bag of 0.9% saline was connected to each hemodialysis and/or hemofiltration device using settings suitable for a 4 kg baby. The saline was suspended over a precise weigh-scale, which was used to continuously monitor fluid movement. As before, we performed short (15-min) and longer (4-h) studies, both with the UF rates set at zero and 40 mL/h, and in the short runs we also applied either no resistance to the access lines, or we added resistance sufficient to generate line pressures of between ± 150 and ± 300 mmHg to the inflow or the outflow lines. In the 15-min tests we undertook four repeat studies under each UF and line-resistance setting (24 runs altogether) for each treatment modality available for that device, and in the 4-h studies we completed two studies for each modality. During the 15-min tests we measured the UF data at the start and end, and during the 4-h tests, we measured it every 5 min. As before, at each data collection we noted three UF values; the rate set on the device, the volume that the device displayed it had achieved, and the amount of fluid it had removed, detected by changes in the weight of the saline bag. This allowed us to calculate both the setting vs. actual errors, and the display vs. actual UF errors, and we report both in relation to the devices. This means that a positive UF error means that the ‘baby’ lost more fluid than was set or displayed (risking fluid depletion in a real-life setting), and a negative one means that the ‘baby’ was delivered extra unrecognised fluid (risking fluid accumulation).

### Devices tested

The PrisMax was tested in the hemofiltration (HF) mode (commonly referred to as continuous veno-venous hemofiltration; CVVH), in the HD mode (commonly referred to as continuous veno-venous hemodialysis; CVVHD), and in the combined hemofiltration and hemodialysis (HDF) mode (commonly referred to as continuous veno-venous hemodiafiltration; CVVHDF). As in the previous Prismaflex HF study, we used the HF20 filter, which has a membrane surface area of 0.2 m^2^, and a total circuit volume of 60 mL. The CARPEDIEM does not support HDF, but was tested in HF and HD modes, using an HCD025 filter with a membrane surface area of 0.29 m^2^, and a total circuit volume of 41 mL. For both devices, the ‘blood’ flow was 50 mL/min, and the fluid replacement and dialysis fluid flow rates were 120 mL/h in HF and HD. For the PrisMax in HDF mode, flows were set at 100 mL/h for both replacement and dialysate fluids. For comparison, the NIDUS has a filter with a surface area of 0.045 m^2^, a standard total circuit volume of 14.8 mL, operates at a blood flow of 20 mL/min, and was tested with a dialysate flow of 400 mL/h.

### Statistics

As well as the newly generated results, we have also included the data for the NIDUS and for the Prismaflex devices collected previously for comparison. We decided not to include the Aquarius machine since the results of our previous study did not warrant further comparisons. In the 15 min studies we used unpaired t-tests to compare different datasets (t), and to determine whether the dataset means differed from zero, and we used the F test to compare their variances (F). We used the statistical package GraphPad Prism v 6.07, and took p values of < 0.05 to indicate a statistically significant difference. We plotted these data with means and 1SD error bars, and tabulated their 5 th, 50 th and 95 th centiles. In the four-hour studies we have simply plotted the results for visual comparison. We also compared the relationships between the setting and display errors for each device in each modality by visualising Bland–Altman plots of the differences between the errors versus their mean values, and did this for the 2022 NIDUS data, as we did not undertake this analysis then.

## Results

Both devices were easy to use, with a very useful visual guidance through the priming phase in the PrisMax.

### The 15-min sessions

**Fig. 1:**
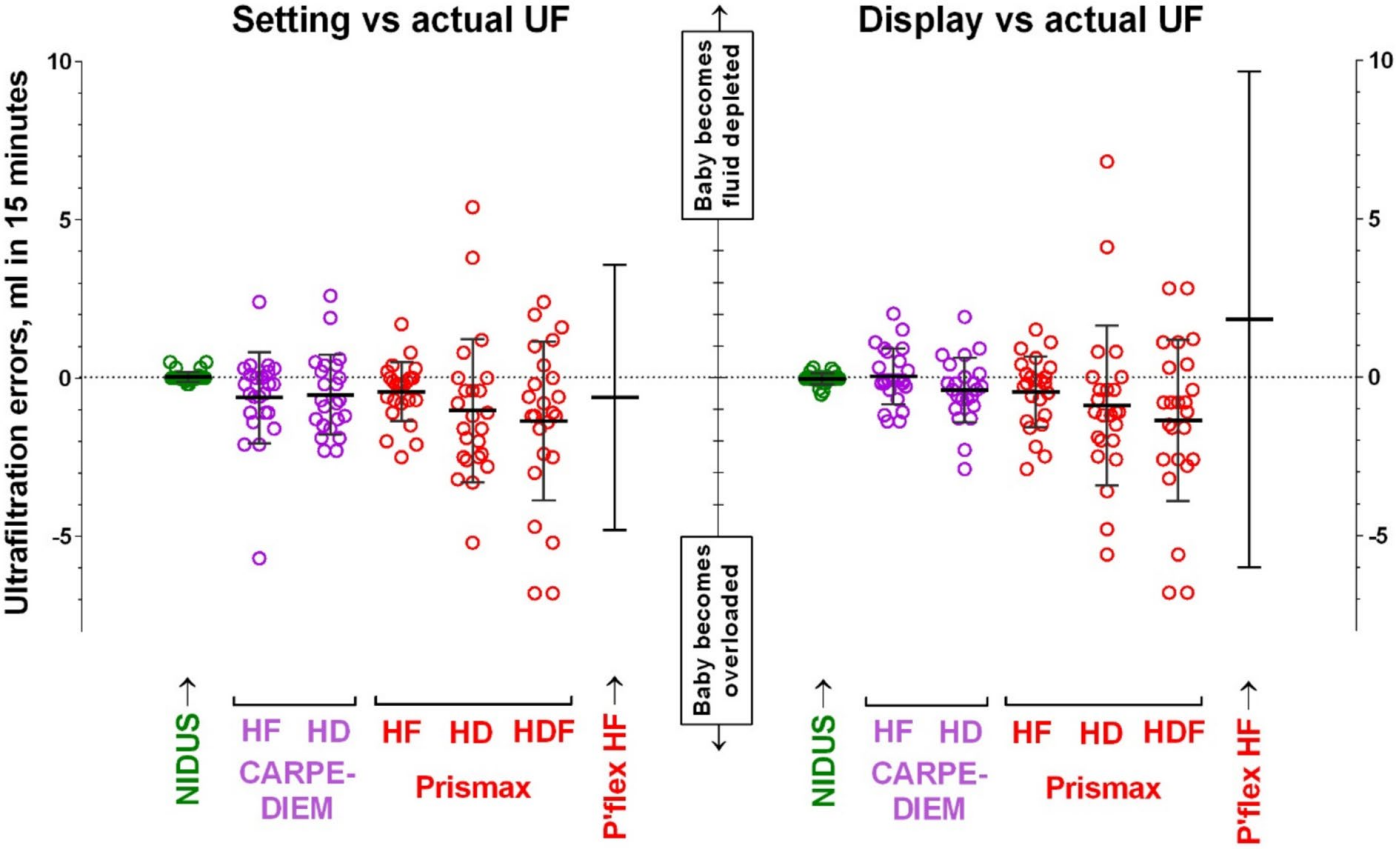
In vitro measurements of ultrafiltratrion (UF) imprecision produced by four hemodialysis and/or hemofiltration devices, measured over 15-min intervals, and expressed as mL per 15 min. The left panel shows the setting vs. actual UF achieved errors, and the right panel shows the display vs. actual UF errors. The error bars indicate mean ± 1SD. The individual data points are not plotted for the Prismaflex as they fall outside of the y-axis limits. HF, hemofiltration (CVVH); HD, hemodialysis (CVVHD); HDF, hemodiafiltration (CVVHDF)

For each individual study, there were no differences between the mean UF values, or their variances, according to whether they were set to maintain stable fluid balance (UF of zero), or to remove fluid at 40 mL/h. Also, there were no differences in the UF data when resistances were applied to either the withdrawal or return line. Therefore, we plotted the combined results for each device-modality combination. Figure 1 shows the plots of the two types of UF error (settings vs. actual, and display vs. actual) for each device, according to the mode of therapy. The means of both types of UF error tended to be slightly negative (less fluid being removed) for the CARPEDIEM and PrisMax studies, but this only reached statistical significance when the PrisMax was delivering HDF (t: p = 0.01 for setting; p = 0.02 for display).

The magnitude of UF errors differed between different devices and modalities, and Table 1 shows their 5 th, 50 th and 95 th centile values (mL difference within 15 min). The variances for both types of error produced by the CARPEDIEM in HF and HD are similar to each other (F: p > 0.2 in each case), but are higher than that generated by the NIDUS (F; p < 0.0001 in each case), and very similar to the variances produced by the PrisMax in its HF modality (F: p = 0.1 for settings; p = 0.4 for display). The variances for both types of error generated by the PrisMax in HD and HDF modes are similar to each other (F: p > 0.6 in each case), but higher than when it is used in HF mode (F: p < 0.0001 for settings errors in both modalities; p = 0.002 for display errors in both modalities). Both types of error generated by the Prismaflex in HF mode were so large that we only plotted the error bars in Fig. 1 as the individual data points were too dispersed to fit onto a convenient graph scale, and their variances were statistically larger than those produced by the PrisMax in any mode (F: p < 0.0001 in each case).

**Table 1:** The 5 th, 50 th and 95 th centile values for the setting vs. actual and the display vs. actual ultrafiltratrion (UF) errors for the NIDUS, CARPEDIEM, Prismax and Plasmaflex (P’flex) devices, according to their treatment modality (mL difference per 15 minutes). HF, hemofiltration (CVVH); HD, hemodialysis (CVVHD); HDF, hemodiafiltration (CVVHDF

Type error	Centile	NIDUS	CARPEDIEM		PrisMax			P'flex
		HD	HF	HD	HF	HD	HDF	HF
Setting vs. actual	5	− 0.2	− 4.8	− 2.3	− 2.4	− 4.7	− 6.8	− 9.5
	50	0.00	− 0.4	− 0.7	− 0.2	− 1.4	− 1.2	− 0.9
	95	0.5	1.19	2.5	1.5	5.0	2.3	7.0
Display vs. actual	5	− 0.5	− 1.4	− 2.8	− 2.8	− 5.4	− 6.8	− 11.0
	50	− 0.05	− 0.1	− 0.4	− 0.2	− 1.1	− 1.0	1.2
	95	0.3	1.9	1.7	1.4	6.1	2.8	17.9

Bland–Altman plots of the setting minus display errors against the mean errors for NIDUS, CARPEDIEM, and PrisMax (Supplementary Fig. 1) showed that in each case their paired values appear to vary randomly in each treatment modality, with the data points mainly clustered around the 0–0 intersections. We did not analyse the Prismaflex or Aquarius data in this way as the magnitude of their errors make them unsuitable for use in infants.

### The four-hour sessions

**Fig. 2:**
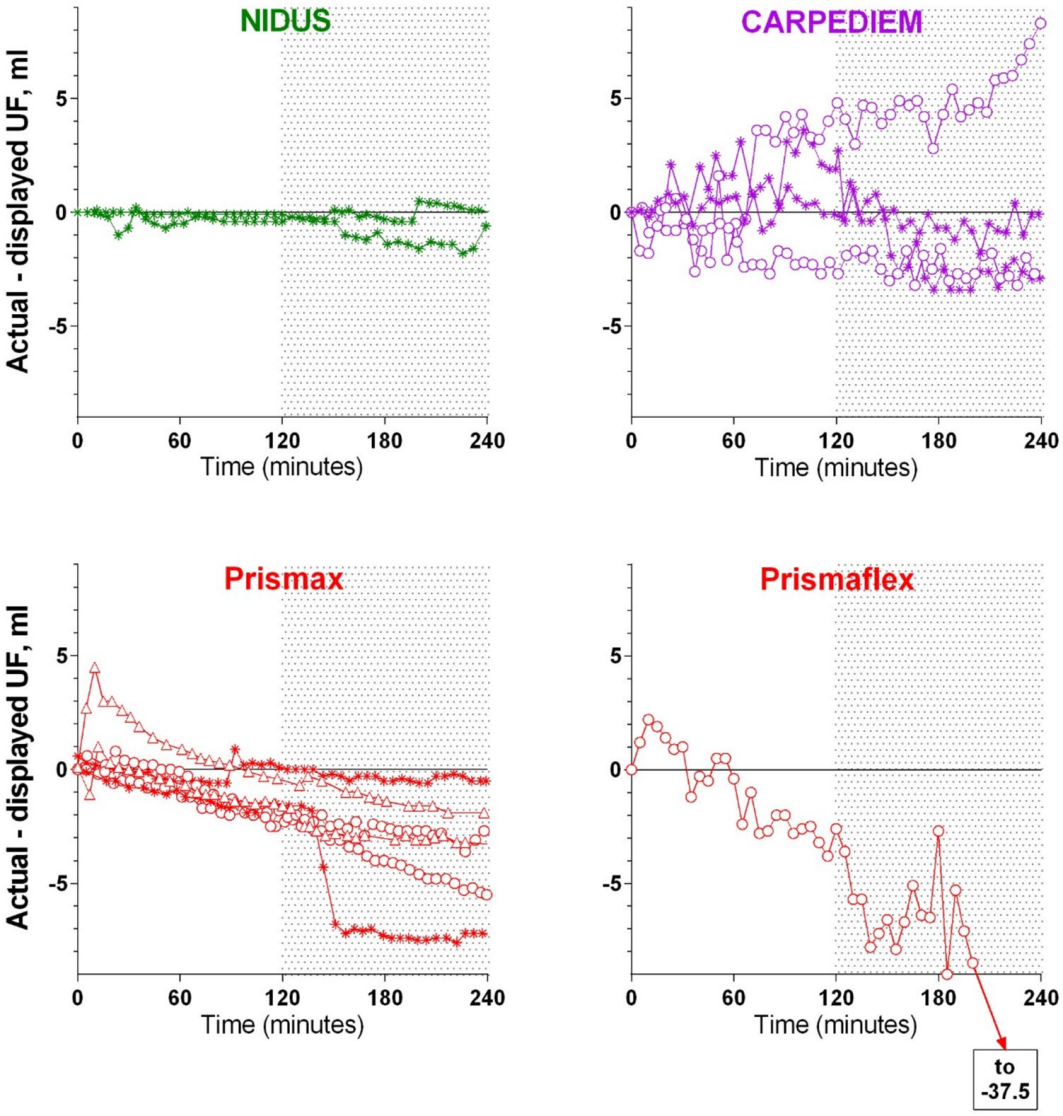
In vitro measurements of ultrafiltratrion (UF) delivered by four hemodialysis and/or hemofiltration devices over 4 h, set to maintain fluid neutrality for the first 2 h, and then to UF at 40 ml/h (shaded area). The y-axes show the difference between the machine’s UF display and the actual UF volume obtained. The stars indcate hemofiltration (HF or CVVH), circles indicate hemodialysis (HD or CVVHD), and triangles indicate hemodiafiltration (HDF or CVVHDF). The value for the Prismaflex UF reached − 37.5 ml by 4 h

Figure 2 shows the cumulative display vs. actual UF errors which occurred during the 4-h ‘treatment’ sessions with each of the devices, with them being set to maintain fluid balance (zero UF) during the first 2 h, and to remove 40 mL/h over the second 2 h (shaded). Departures from zero represent the difference between the information presented to the clinician and the true fluid balance. The HD sessions are shown as stars, the HF sessions as open circles, and the HDF sessions as open triangles. The plots for the NIDUS and the Prismaflex are the same as in our previous publication [2], with NIDUS having a maximum discrepancy of 2.6 mL/4 h, and final differences of 0.1 and − 0.5 mL/4 h, and Prismaflex reaching a final discrepancy of − 37.5 mL by 4 h. The CARPEDIEM and PrisMax discrepancies fell between those of the NIDUS and the Prismaflex, with final errors in the range of ± 9 mL/4 h, with 9 of the 10 runs removing less fluid than the device displayed. Comparing these curves to the 15 min results suggests that the devices are operating over time to minimise the cumulative deviation of the errors, with the overall discrepancies mostly accruing relatively ‘smoothly’ rather than in sudden jumps.

## Discussion

We have shown that the accuracy and precision of the UF control of the CARPEDIEM and PrisMax are similar in the two devices, and substantially better than the Prismaflex which we tested previously. Most of the discrepancies between the set or displayed fluid volumes and the actual volumes generated were within about ± 6 mL over 15 min, but the cumulative errors only rose to within about ± 9 mL by the end of the four-hour treatment sessions, regardless of whether the treatment modality included HF, HD, or both. For the CARPEDIEM, this degree of imprecision is higher than the developers’ claim of 1 g/h [3]. Assuming an infant’s blood volume to be 80 mL/kg, this degree of imprecision could result in about a 4.5% change in the circulatory volume of a 2.5 kg baby (the lower end of the weight range from the manufacturer’s marketing information), which may or may not be tolerated depending on their clinical stability. There is a tendency for both devices to ‘undertreat’ with respect to fluid removal, which means that their errors would be slightly more likely to leave them with fluid accumulation, rather than hypovolaemic or dehydrated. NIDUS is the only device to have a highly precise fluid control system, achieved by using a volumetrically-controlled syringe-driven circuit [2, 4], making it theoretically safer for treating unstable babies and those < 3 kg.

If the extracorporeal circuit volume should not exceed 10% of the blood volume, using these devices would also require blood-priming for babies of < 4 kg for the CARPEDIEM using its smallest currently available filter option (circuit 32 mL), and for those weighing < 7.5 kg for the PrisMax (circuit 60 mL). The PrisMax has the disadvantage of requiring blood priming over a wider range of patient weights, and the two advantages of advanced software, and the capacity to be used to treat much larger children. The standard NIDUS circuit volume of 14.8 mL does not need priming for babies weighing 1.9 kg or greater, and this volume can be reduced to 9.8 ml by using a smaller stroke volume, when it only needs priming for treating babies < 1.2 kg. In real-world clinical use, NIDUS delivered more accurate fluid removal than controls (who were treated with Prismaflex, or Aquarius, or peritoneal dialysis) in babies weighing between 1.0 and 7.8 kg, in a prospective, randomised, controlled, multicentre clinical trial in paediatric intensive care units across the UK, where UF losses were measured by weighing [4]. Although the same precise fluid treatment could be delivered by hand-operating a circuit constructed from syringes, a filter, and some 3-way taps [5, 6], we found it to be a labour-intensive, highly repetitive task, where it was sometimes difficult for staff to maintain safe levels of concentration over long treatment periods [7].

At present, as far as we are aware, no regulatory authorities require manufacturers to provide evidence of the accuracy or precision of UF control for devices designed to deliver HF or HD to small children. The in vitro testing we undertook is simple to perform and can provide a useful guide to a lower weight cut-off of children likely to be adversely affected by imprecision by comparing the size of potential fluid imbalance with blood volume. We recommend that this type of testing should be adopted as routine by regulatory authorities and manufacturers. Previous reports have expressed concerns about using Prismaflex for small children [1], and our testing does not challenge that. The PrisMax and the CARPEDIEM are likely to be more precise for infants, and the NIDUS is both accurate and efficacious for treating babies from 800 g to 8 kg and may be particularly suited to unstable and smaller babies.

An important shortcoming of our vitro testing method is that we use saline rather than blood. It is likely that the accuracy and precision data will apply in vivo, but the finding that a device can successfully ultrafilter 40 mL/h of saline does not necessarily imply that these rates would be achievable in every clinical setting. We have not yet had the opportunity to evaluate the UF precision of the Aquadex device which is used off-license in the US [8], but hope that future collaboration with clinical teams and the manufacturers might achieve this. We used arbitrary blood and fluid flow rates for the different devices according to clinical judgement and default rates, but do not believe that this will have impacted the ultrafiltration results measured.

## Conclusion

This in vitro study has shown that the PrisMax has a greatly improved precision of UF control compared to its predecessor, the Prismaflex, with fluid-balance errors of < 9 mL in a four-hour treatment. This performance is matched by the CARPEDIEM, which was previously claimed to have UF control precise to < 1 mL/h. The only device capable of delivering ultra-precise UF is the NIDUS, which is still undergoing regulatory scrutiny/approval. Thus, kidney replacement for small babies remains an unmet area of clinical need which urgently needs to be addressed.

## Supplementary Information

Below is the link to the electronic supplementary material.

Graphical abstract (PPTX 248 KB)

Supplementary file1 (PDF 243 KB)

## Data Availability

The data from this study are available on reasonable request.
