# Supplementary material for: In vitro measurements of ultrafiltration precision in hemofiltration and hemodialysis devices used in infants, Part 2: Comparison of PrisMax and CARPEDIEM with previous data on NIDUS, Prismaflex and Aquarius
Source: Pediatr Nephrol. 2025 Jul 8;40(11):3549–54. doi: 10.1007/s00467-025-06788-0 (PMC12484341; doi:10.1007/s00467-025-06788-0)
Supplement: Supplementary file 1 — Graphical abstract (PPTX 248 KB) [file 467_2025_6788_MOESM1_ESM.pptx]

## Slide 1
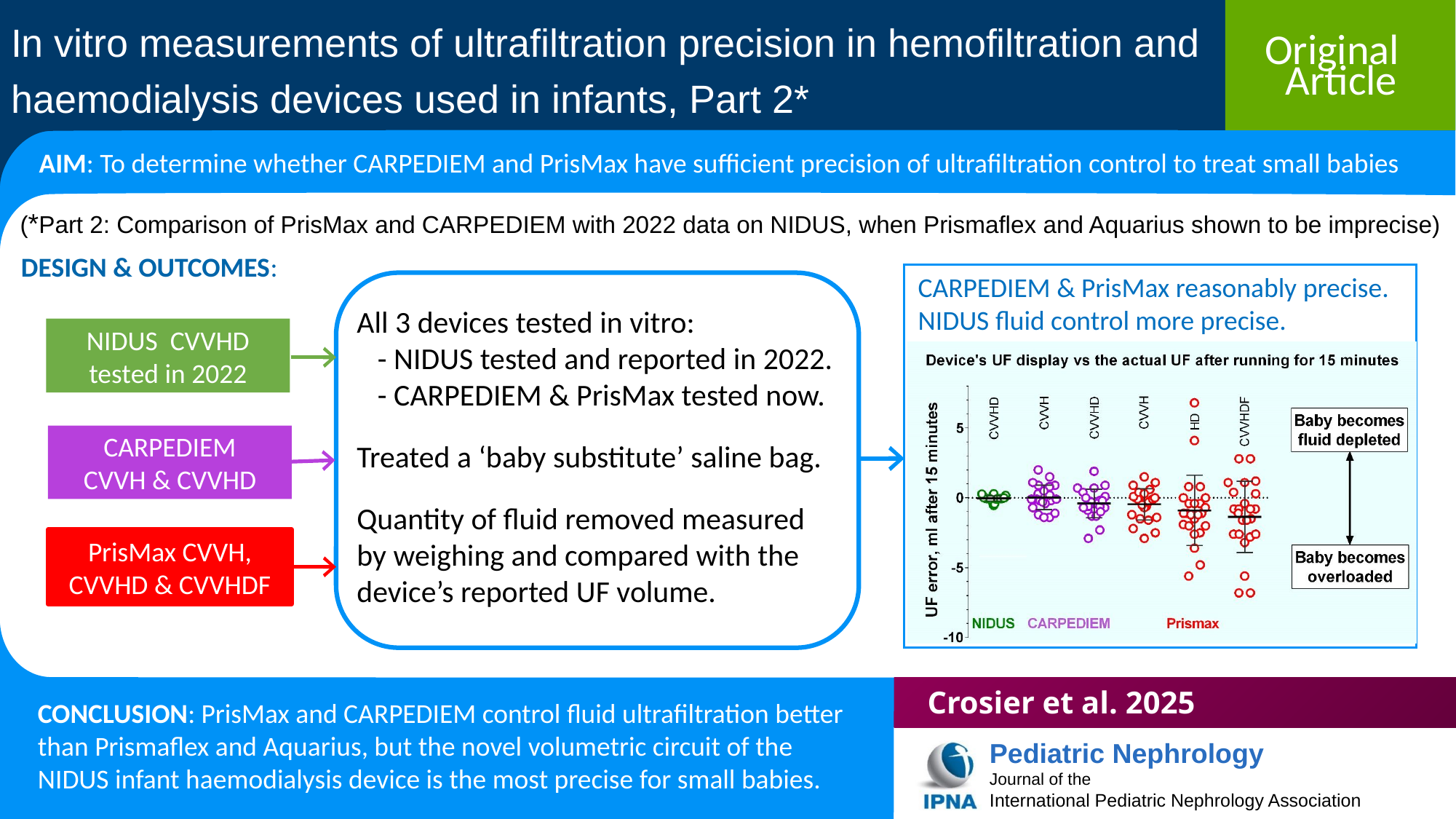

In vitro measurements of ultrafiltration precision in hemofiltration and haemodialysis devices used in infants, Part 2*
AIM: To determine whether CARPEDIEM and PrisMax have sufficient precision of ultrafiltration control to treat small babies
(*Part 2: Comparison of PrisMax and CARPEDIEM with 2022 data on NIDUS, when Prismaflex and Aquarius shown to be imprecise)
DESIGN & OUTCOMES:
CARPEDIEM & PrisMax reasonably precise.
NIDUS fluid control more precise.
All 3 devices tested in vitro:
 - NIDUS tested and reported in 2022.
 - CARPEDIEM & PrisMax tested now.
Treated a ‘baby substitute’ saline bag.
Quantity of fluid removed measured by weighing and compared with the device’s reported UF volume.
NIDUS CVVHD tested in 2022
CARPEDIEM
CVVH & CVVHD
PrisMax CVVH,
CVVHD & CVVHDF
Crosier et al. 2025
CONCLUSION: PrisMax and CARPEDIEM control fluid ultrafiltration better than Prismaflex and Aquarius, but the novel volumetric circuit of the NIDUS infant haemodialysis device is the most precise for small babies.
