# Supplementary material for: In vitro measurements of ultrafiltration precision in hemofiltration and hemodialysis devices used in infants, Part 2: Comparison of PrisMax and CARPEDIEM with previous data on NIDUS, Prismaflex and Aquarius
Source: Pediatr Nephrol. 2025 Jul 8;40(11):3549–54. doi: 10.1007/s00467-025-06788-0 (PMC12484341; doi:10.1007/s00467-025-06788-0)

Supplementary Figure 1 Bland-Altman graphs of setting error minus display error against the mean of the errors for the 15-minute studies of NIDUS being used in hemodialysis (HD) mode, of CARPEDIEM being used in the HD and hemofiltration (HF) mode, and of PrisMax being used in HF, HD and hemodiafiltration (HDF) modes.

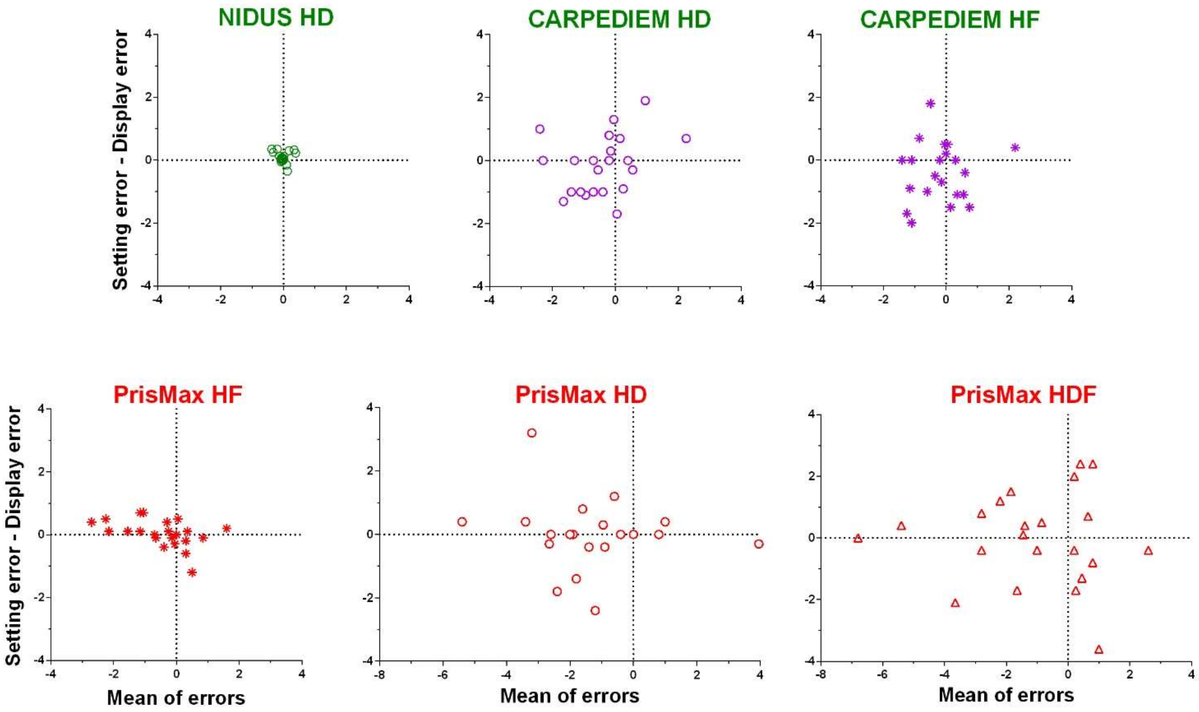

Supplement: Supplementary file 2 — Supplementary file1 (PDF 243 KB) [file 467_2025_6788_MOESM2_ESM.pdf]
